# Gene expression signature in mouse thyroid tissue after ^131^I and ^211^At exposure

**DOI:** 10.1186/s13550-015-0137-8

**Published:** 2015-10-22

**Authors:** Nils Rudqvist, Johan Spetz, Emil Schüler, Britta Langen, Toshima Z. Parris, Khalil Helou, Eva Forssell-Aronsson

**Affiliations:** Department of Radiation Physics, Institute of Clinical Sciences, Sahlgrenska Cancer Center, Sahlgrenska Academy, University of Gothenburg, SE-413 45 Gothenburg, Sweden; Department of Oncology, Institute of Clinical Sciences, Sahlgrenska Cancer Center, Sahlgrenska Academy, University of Gothenburg, SE-413 45 Gothenburg, Sweden

**Keywords:** Radiation biology, Microarray, Radiation biomarkers, Radionuclide therapy, Transcriptomics, Radiogenomics

## Abstract

**Background:**

^131^I and ^211^At are used in nuclear medicine and accumulate in the thyroid gland and may impact normal thyroid function. The aim of this study was to determine transcriptional profile variations, assess the impact on cellular activity, and identify genes with biomarker properties in thyroid tissue after ^131^I and ^211^At administration in mice.

**Methods:**

To further investigate thyroid tissue transcriptional responses to ^131^I and ^211^At administration, we generated a new transcriptional dataset that includes re-evaluated raw intensity values from our previous ^131^I and ^211^At studies. Differential transcriptional profiles were identified by comparing treated and mock-treated samples using Nexus Expression 3.0 software. Further data analysis was performed using R/Bioconductor and IPA.

**Results:**

A total of 1144 genes were regulated. Hierarchical clustering subdivided the groups into two clusters containing the lowest and highest absorbed dose levels, respectively, and revealed similar transcriptional regulation patterns for many kallikrein-related genes. Twenty-seven of the 1144 genes were recurrently regulated after ^131^I and ^211^At exposure and divided into six clusters. Several signalling pathways were affected, including calcium, integrin-linked kinase, and thyroid cancer signalling, and the peroxisomal proliferator-activated receptor network.

**Conclusions:**

Substantial changes in transcriptional regulation were shown in ^131^I and ^211^At-treated samples, and 27 genes were identified as potential biomarkers for ^131^I and ^211^At exposure. Clustering revealed distinct differences between transcriptional profiles of both similar and different exposures, demonstrating the necessity for better understanding of radiation-induced effects on cellular activity. Additionally, ionizing radiation-induced changes in kallikrein gene expression and identified canonical pathways should be further assessed.

## Background

Medical applications for radionuclides are rapidly developing. The β particle-emitting ^131^I is frequently included in therapy regimens of various thyroid disorders due to selective uptake of the isotope in thyroid tissue and is also administered bound to tumour-seeking agents for therapeutic and diagnostic purposes [1–4]. The α particle-emitting ^211^At is a suitable therapeutic radionuclide due to, e.g., a nearly optimal therapeutic linear energy transfer value of emitted α particles of 98.8 keV/μm [5]. ^211^At-labelled tumour-seeking pharmaceuticals have been utilized both in humans and in animals [6–8]. Similar to unbound ^131^I, selective uptake of unbound ^211^At also occurs in thyroid tissue [9–11], and administration of free ^131^I and ^211^At or ^131^I- and ^211^At-labelled radiopharmaceuticals have been shown to result in thyroid irradiation [6, 12]. Additionally, nuclear accidents often involve an atmospheric release of ^131^I, as was the case in connection with the Chernobyl accident, which resulted in an increased incidence of thyroid cancer in children [13, 14].

Despite the risk of exposing thyroid tissue to ^131^I and ^211^At, the understanding of radiation-induced effects is far from complete and molecular biomarkers of absorbed dose or radiation-induced effects on thyroid tissue are yet to be identified. Biomarkers are useful to indicate achieved therapeutic effects or estimate risk exposure and evaluate the quality and severity of side effects. RNA microarray analysis is a semi-quantitative method to identify changes in genome-wide transcriptional patterns between two or more samples. The result is a transcriptional profile, i.e. a snapshot of the radiation-induced cellular activity at the mRNA level. This can be used to determine the impact of radiation on biological functions and canonical pathways, to predict upstream regulation of target molecules, and for biomarker discovery without the risk of bias in focusing on a specific set of signalling pathways only.

Few investigations on global gene expression effects of α and β particle irradiation have been performed in normal (thyroid) tissue in vivo. There are, however, a few in vitro studies on global gene expression in fibroblasts and cancer cells after α particle exposure [15, 16]. Additionally, effects on gene expression for a set of pre-defined genes after α particle irradiation have been measured both in cancer cells in vitro and in vivo in xenografted tumours [17–19]. Previously, we have published results showing substantial differences between transcriptional profiles in thyroid tissue in vivo after different ^131^I or ^211^At exposures, varying absorbed dose, dose rate and time after administration [20–22]. We then identified potential biomarkers for each type of exposure separately and concluded that biological response to radiation is complex and that it is difficult to predict or extrapolate radiation-induced effects for other exposure parameters.

The aim of this work was to re-evaluate the previously obtained transcriptional response in thyroid after administration of ^131^I^−^ and free ^211^At in mice from different exposure conditions to gain a better understanding of variations in transcriptional regulation on absorbed dose, dose rate, time after administration and radiation quality.

## Methods

### Study design

In this study, we further investigate the thyroid transcriptional response to ^131^I and ^211^At exposure by using normalized intensity values from three different experiments where separate analyses of each experiment have been published elsewhere [20–22]. All normalized intensity values (normalization was performed according to experiment) from these three experiments were imported together into Nexus Expression 3.0 (BioDiscovery; El Segundo, CA) for filtering, linear modelling and determination of differentially expressed genes.

No new tissue sampling and analysis was thus performed in this study, but a reanalysis of all these data together in a new analysis by Nexus Expression. Data presented in the present work are therefore novel and have not been previously published.

A brief summary of the methods used in the three original studies is as follows: a total of 44 female Balb/c nude mice (CAnN.Cg-Foxn1nu/Crl, Charles River Laboratories International, Inc., Salzfeld, Germany) (*n* = 2–3 per group) were i.v. injected with various amounts of ^131^I or ^211^At in the tail vein and killed at 1, 6, 24 or 168 h after administration, or mock-treated (Table 1). Thyroid, kidney, liver, lung and spleen tissue samples were collected and immediately snap-frozen in liquid nitrogen and stored at −80 °C until RNA extraction. The present study contains data on transcriptional changes in thyroid glands. The transcriptional response in the kidneys, livers, lungs and spleens has been published elsewhere [23–25].

**Table 1:** Number of regulated genes in thyroid tissue in mice 1–168 h after administration of ^131^I and ^211^At

Radionuclide	Δ*t* (h)	A (kBq)	D (Gy)	Mice (*n*)	Regulated genes (no.)		
					Total	Up	Down
^211^At	1	1.7	0.023	3	210	92	118
		100	1.4	3	630	345	285
	6	1.7	0.32	3	170	51	119
		7.5	1.4	3	290	116	174
	24	0.064	0.05	3	360	254	106
		0.64	0.5	3	157	95	62
		1.7	1.4	3	359	149	210
		14	11	3	464	339	125
		42	32	3	357	251	106
	168	1.7	1.8	3	136	49	87
^131^I	24	13	0.85	2	227	101	126
		130	8.5	2	266	138	128
		260	17	2	55	37	18

Absorbed dose was calculated using MIRD formalism

*Abbreviations*: *Δt* exposure time, *A* injected activity, *D* absorbed dose

The methods for determination of absorbed doses have been reported previously with separate analysis of each experiment [20–22]. In short, the mean absorbed dose was calculated according to conventional Medical Internal Radiation Dose (MIRD) formalism. We used previously published data on relative activity concentration of ^131^I and ^211^At in thyroid in mice [26], an absorbed fraction of 0.742 and 1 for radiation emitted from ^131^I and ^211^At, respectively [27], and a standard mouse thyroid mass of 3 mg.

### Gene expression analysis

Genome-wide transcriptional analysis using RNA microarray of thyroid tissue has been described elsewhere [20, 21]. Briefly, RNA samples were analysed using MouseRef-8 Whole-Genome Expression Beadchips (Illumina; San Diego, CA, USA). Nexus Expression 3.0 (BioDiscovery; El Segundo, CA) was used to identify statistically significant differentially expressed transcripts (≥1.5-fold change) with a Benjamini-Hochberg adjusted *p* value cut-off of 0.01 between irradiated and control tissues. RNA microarray data from irradiated samples were compared with RNA microarray data from control animals from each separate experiment to ensure that differential expression of genes reflects radiation-induced changes and not variations between different control groups. There was one difference in study design between the present and previous studies. In the present work, data from tissue samples from animals administered 0.064 and 0.64 kBq ^211^At and killed 24 h after administration were compared with data from tissue samples from control animals killed the same day. However, in the previous paper with analysis of the transcriptional response at 24 h after ^211^At administration, animals administered 0.064 and 0.64 kBq were compared with controls killed earlier (killed simultaneously as animals injected with 1.7, 14 and 42 kBq, also at 24 h after administration but on another day) [20]. In the present paper, the term “regulated transcripts/genes” is used synonymous to “statistically significant differentially expressed transcripts/genes”.

Hierarchical clustering of regulated transcripts according to their transcriptional regulation profile was performed using the hclust function (stats package, version 3.1.1) with the complete linkage algorithm and Lance-Williams dissimilarity update formula in the R statistical computing environment (version 0.97.551, http://www.r-project.org) [28]. Heat maps were produced using the heatmap.2 function (gplots package, version 2.14.2).

Upstream regulation, diseases and functions and canonical pathway analyses were generated using the Ingenuity Pathway Analysis tool (IPA, Ingenuity® Systems, www.ingenuity.com; Redwood City, CA) with Fisher’s exact test (*p* value <0.05).

Gene expression data discussed in this publication have been deposited at the NCBI’s Gene Expression Omnibus (GEO accession numbers: GSE32306 [20], GSE54594 [21] and GSE66089 [22]).

## Results

### Regulated genes

**Fig. 1:**
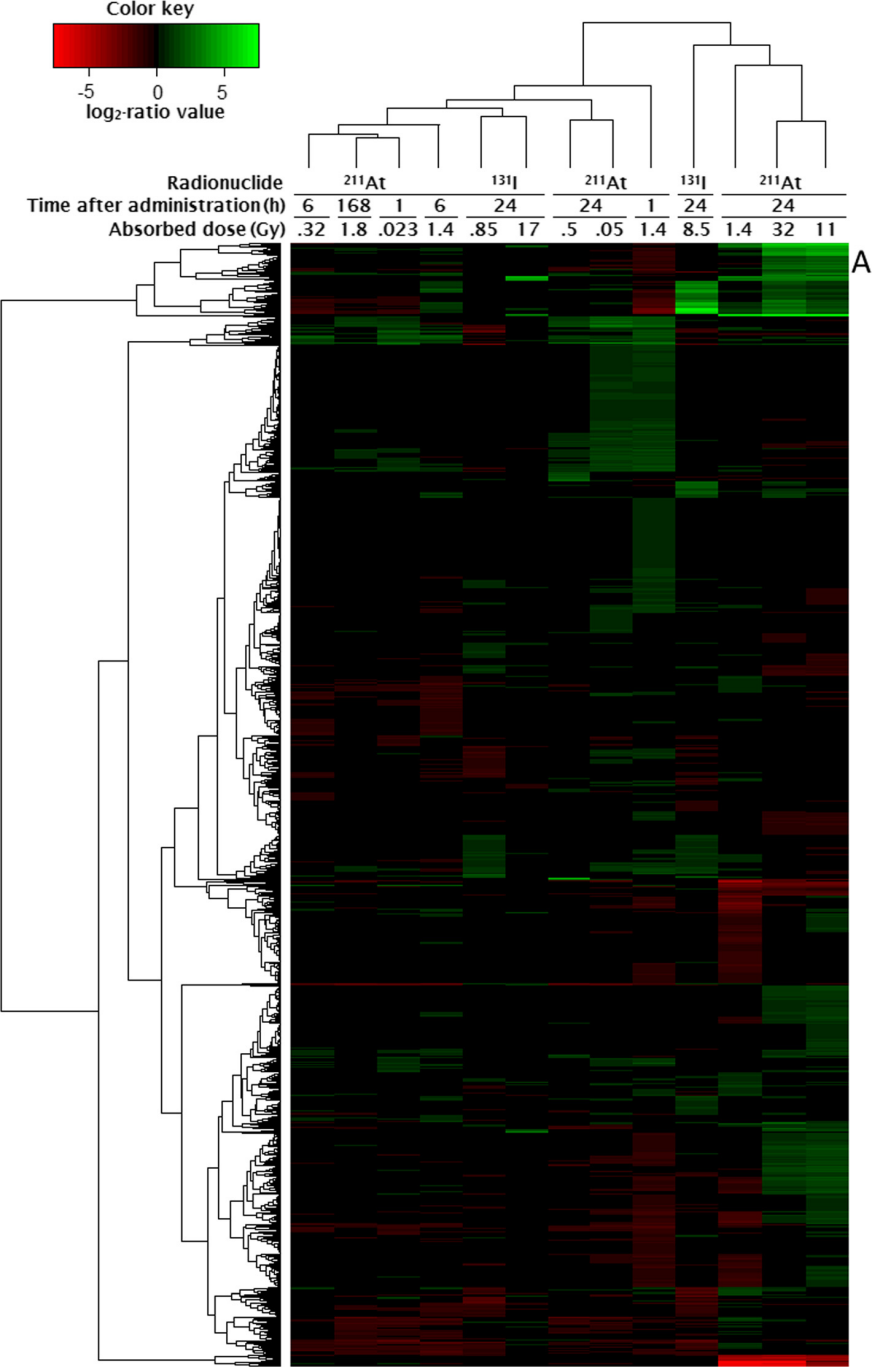
Results from hierarchical clustering analyses of genes and exposure groups after ^131^I and ^211^At exposure. Genes were hierarchically clustered according to log_2_-ratio values using gplots package in R/Bioconductor. The *columns* represent genes and *rows* represent exposure type. Cluster containing kallikrein genes is denoted *A*

In the present study, 1144 genes (1164 transcripts) were regulated. The number of regulated genes in each group varied between 55 (17 Gy, 24 h, ^131^I) and 630 (1.4 Gy, 1 h ^211^At) (Table 1). Hierarchical clustering subdivided the exposure groups into two larger clusters: one smaller branch containing groups with higher absorbed dose levels (1.4–32 Gy from ^211^At and 8.5 Gy from ^131^I at 24 h) and a larger branch with the remaining groups (Fig. 1).

At both 1 h and 6 h after ^211^At administration, a higher absorbed dose/dose rate resulted in a higher number of regulated genes (Table 1). In contrast, at 24 h after ^211^At administration, the number of regulated genes varied non-monotonously with absorbed dose. A slight increase in the number of genes regulated after ^131^I exposure was seen between 0.85 and 8.5 Gy and a decrease between 8.5 and 17 Gy. Transcriptional profiles at 1, 6 and 168 h clustered together, with the exception of 1.4 Gy at 1 h (Fig. 1). At 24 h, the transcriptional profiles for 0.05 and 0.5 Gy clustered together while the profiles for 1.4, 11 and 32 Gy clustered together with highest similarity between 11 and 32 Gy. Furthermore, upregulation of 110 genes was shared between 0.05 and 1.4 Gy at 24 and 1 h after ^211^At administration, respectively. For ^131^I exposure, the transcriptional profiles for 0.85 and 17 Gy clustered together, whereas the response after 8.5 Gy was more similar to that after 1.4, 11 and 32 Gy at 24 h following ^211^At administration. In the groups exposed to 1.4 Gy, the number of regulated genes decreased from 630 to 290 between 1 and 6 h, but increased to 359 at 24 h (Table 1). Additionally, the transcriptional profiles of these groups showed little similarity in the cluster analysis (Fig. 1). Lastly, an equal amount of injected activity (1.7 kBq, ^211^At) resulted in 210, 170, 359 and 136 regulated genes at 0.023, 0.32, 1.4 and 1.8 Gy after 1, 6, 24 and 168 h, respectively (Table 1). The transcriptional profiles of these groups were similar and clustered together with the exception of 1.4 Gy at 24 h which was clearly different (Fig. 1).

### Kallikrein 1 and kallikrein 1-related peptidases

**Fig. 2:**
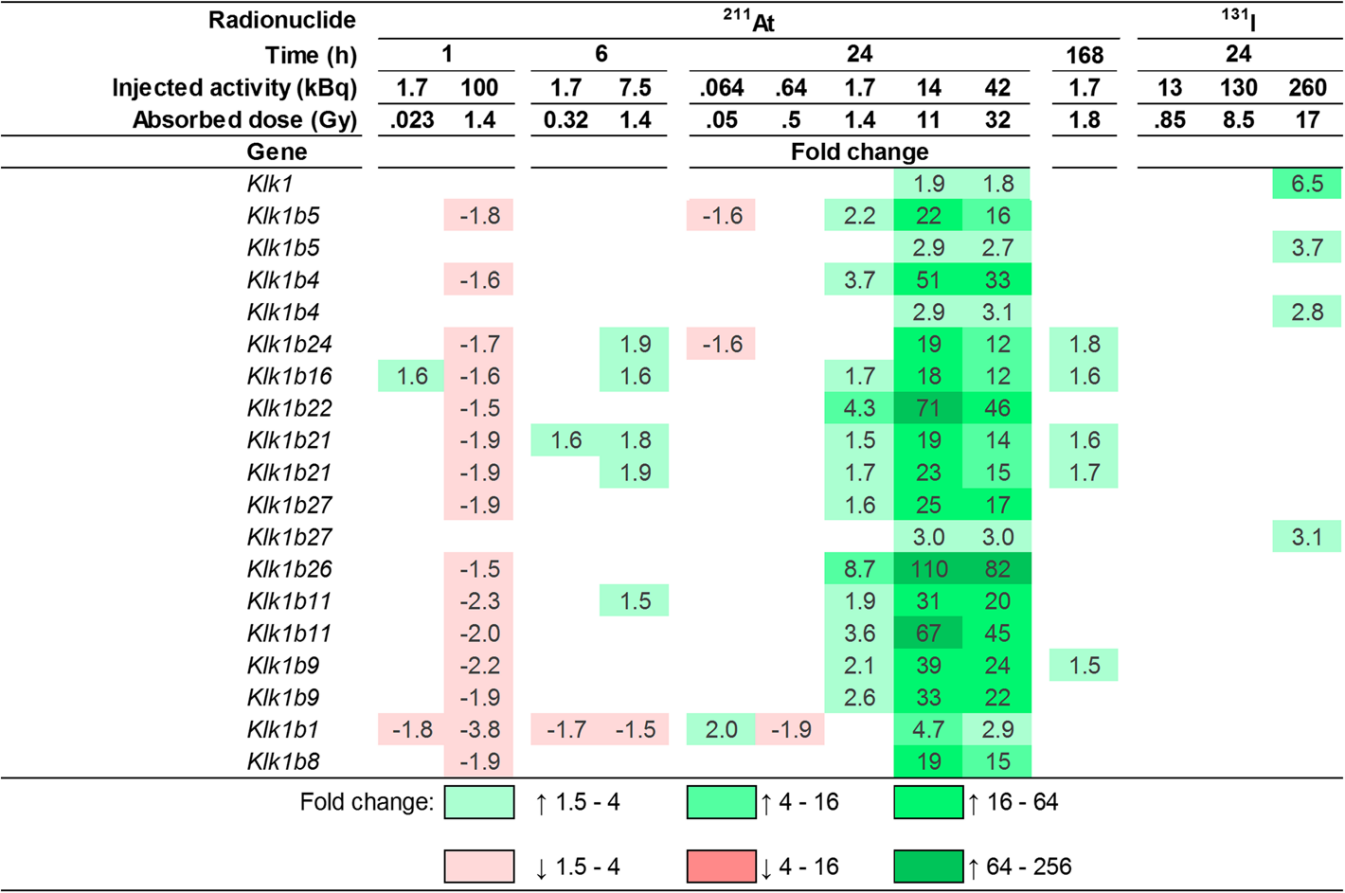
Regulation of 13 kallikrein genes belonging to the peptidase S1 family after ^131^I and ^211^At exposure. Values indicate fold change of differential gene expression. *Green* and *red colours* indicate up- and downregulation of genes, respectively. Higher saturation of colours indicates higher fold change

Hierarchical clustering of all regulated transcripts revealed similar transcriptional regulation profiles for 13 kallikrein genes belonging to the peptidase S1 family (*Klk1*, *Klk1b1*, *Klk1b4*, *Klk1b5*, *Klk1b8*, *Klk1b9*, *Klk1b11*, *Klk1b16*, *Klk1b21*, *Klk1b22*, *Klk1b24*, *Klk1b26* and *Klk1b27* (A in Figs. 1 and 2). All 13 genes were upregulated at 11 and 32 Gy 24 h after ^211^At administration. For an absorbed dose of 1.4 Gy after ^211^At exposure, 9/13 kallikrein genes were upregulated at 24 h but downregulated at 1 h (three additional genes were downregulated at 1 h), while at 6 h, 1 and 4 genes were down- and upregulated, respectively. Furthermore, 4/13 genes were upregulated at 1.8 Gy 168 h after injection of ^211^At. At 24 h after ^131^I administration, 4/13 genes were upregulated after 17 Gy, but not regulated at the lower absorbed dose levels.

### Recurrently regulated genes after ^131^I and ^211^At exposure

**Fig. 3:**
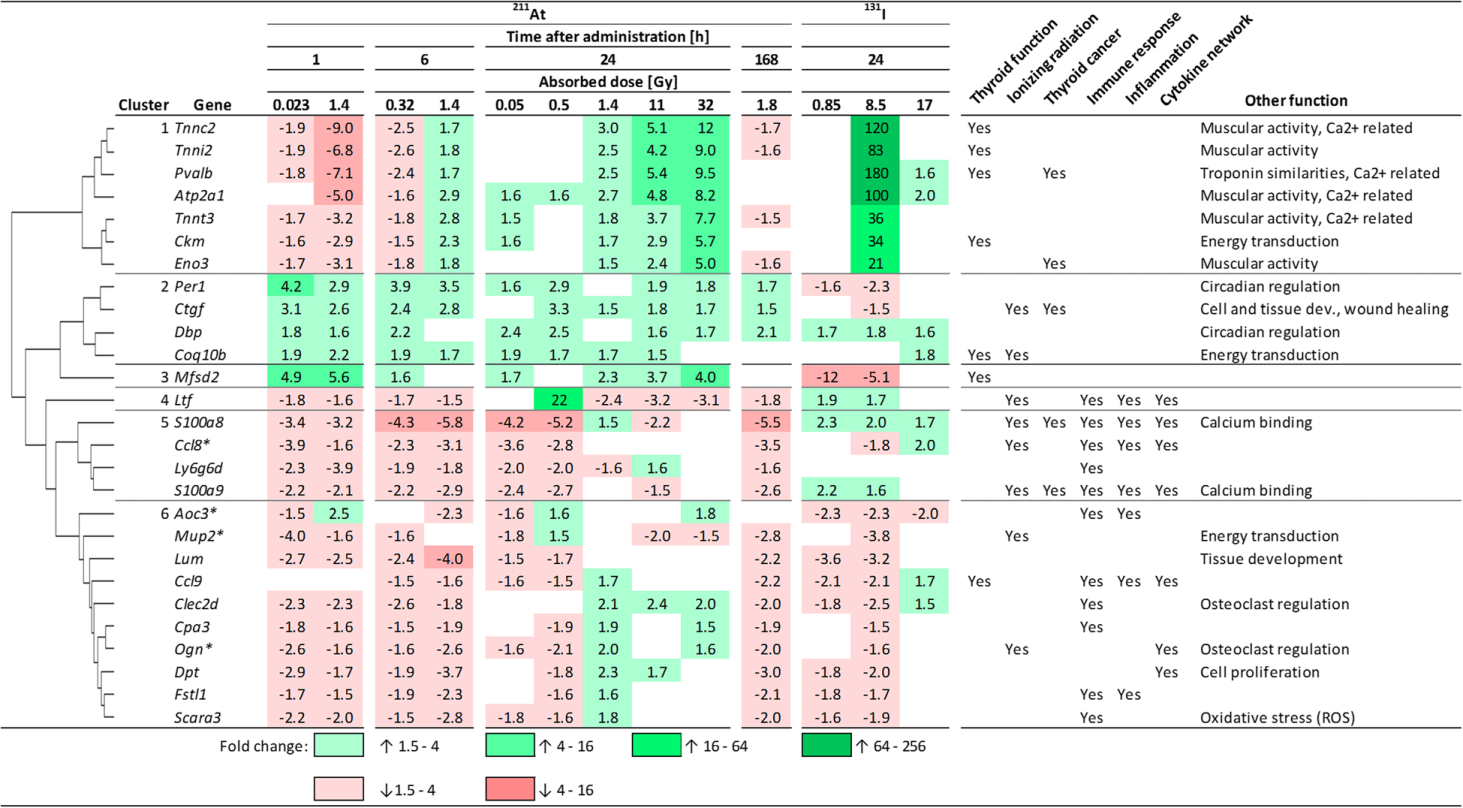
Regulation of 27 recurrently regulated genes after ^131^I and ^211^At exposure. Values indicate fold change of differential gene expression. *Green* and *red colours* indicate up- and downregulation of genes, respectively. Higher saturation of colours indicates higher fold change. The relationships between genes and thyroid function, ionizing radiation, and thyroid cancer and biological function of genes have been assessed using literature reports

Twenty-seven of the 1144 regulated genes were regulated in ≥9 of the 13 groups (Fig. 3). Hierarchical clustering divided the 27 genes into six groups: 1) *Atp2a1*, *Ckm*, *Eno3*, *Pvalb*, *Tnnc2*, *Tnni2*, *Tnnt3*; 2) *Coq10b*, *Ctgf*, *Dbp*, *Per1*; 3) *Mfsd2*; 4) *Ltf*; 5) *Ccl8*, *Ly6g6d*, *S100a8*, *S100a9* and 6) *Aoc3*, *Ccl9*, *Clec2d*, *Cpa3*, *Dpt*, *Fstl1*, *Lum*, *Mup2*, *Ogn*, *Scara3*. In cluster 1, genes were downregulated at 1 h following ^211^At administration. This changed at 6 h where 0.32 and 1.4 Gy showed down- and upregulation, respectively. At 24 h, the regulated genes were upregulated and, notably, the fold change increased with absorbed dose. At 24 h after ^131^I administration, all of these genes were upregulated with high fold changes (21–180) after 8.5 Gy, while two genes were upregulated to low extent after 17 Gy. Genes in cluster 2 were upregulated after ^211^At administration while ^131^I administration resulted in both up- and downregulation. The *Mfsd2* gene, the only gene in cluster 3, was up- and downregulated after ^211^At and ^131^I administration, respectively. The *Ltf* gene, sole gene in cluster 4 with almost opposite regulation to *Mfsd2*, was downregulated after ^211^At administration with the exception of a 22-fold upregulation after 0.5 Gy at 24 h, and upregulated after ^131^I administration. Cluster 5 was similar to cluster 4, and ^211^At administration resulted in downregulation with few exceptions of upregulation at 24 h and ^131^I administration generally resulted in upregulation. Genes in cluster 6 were generally downregulated with the exception of upregulation at, e.g., 1.4–32 and 17 Gy 24 h after ^211^At and ^131^I administration, respectively.

### Canonical pathway analysis: calcium, integrin-linked kinase and thyroid cancer signalling

In the present study, calcium, integrin-linked kinase and thyroid cancer signalling were the top three canonical pathways generated using the IPA comparison analysis tool (Table 2). An impact on calcium signalling was statistically significant in all groups except at the two lowest and the highest absorbed dose levels 24 h after ^211^At and ^131^I administrations, respectively. Integrin-linked kinase signalling was statistically significant in all groups except at low absorbed dose levels at 1 and 24 h, and at the lowest and highest absorbed dose levels 24 h after ^211^At and ^131^I administration, respectively. Genes associated with calcium and integrin-linked kinase signalling were generally downregulated early and at low absorbed doses and upregulated at later time points and at higher absorbed doses. Thyroid cancer signalling was statistically significant in all groups except at 0.023 and 0.05 Gy, 1 and 24 h following ^211^At administration, respectively, and at 0.85 and 8.5 Gy 24 h after ^131^I administration (Table 2). Additionally, the number of genes involved in the different canonical pathways at each time point generally increased with absorbed dose (with exceptions between 11 and 32 Gy).

**Table 2:** Top three Ingenuity canonical pathways enriched by genes regulated after ^131^I or ^211^At exposure

Canonical pathway	Nuclide	Δ*t* (h)	D (Gy)	*p* value	Involved molecules^a^
Calcium signalling	^211^At	1	0.023	0.035	DOWN: MYL1, TNNC2, TNNI2, TNNT3, TPM2
			1.4	0.001	DOWN: ACTA1, ATP2A1, ATP2A3, Calm1 (includes others), MYH1, MYH2, MYH4, MYL1, TNNC2, TNNI2, TNNT3, TPM2 UP: LETM1, PRKACA
		6	0.32	<0.0005	DOWN: ACTA1, ATP2A1, MYH1, MYL1, TNNC2, TNNI2, TNNT3, TPM2
			1.4	<0.0005	DOWN: Calm1 (includes others) UP: ACTA, ATP2A1, ATP2A3, MYH, MYH2, MYH4, RYR1, TNNC2, TNNI2, TNNT3, TPM2
		24	0.05	*0.180*	*UP: ATP2A1, LETM1, MYH2, RYR1, TNNT3*
			0.5	*0.390*	*UP: ATP2A1, MYH2*
			1.4	0.009	DOWN: ATP2A3 UP: ACTA1, ATP2A1, MYH4, TNNC2, TNNI2, TNNT3, TPM2
			11	<0.0005	DOWN: Camk2b, LETM1 UP: ACTA1, ATP2A1, ATP2A3, Calm1 (includes others), CREB3L4, MYH1, MYH2, MYH4, MYL1, RYR1, TNNC2, TNNI2, TNNT3, TP63, TPM2
			32	<0.0005	DOWN: Camk2b, LETM1 UP: ACTA1, ATP2A1, ATP2A3, CREB3L4, MYH1, MYH2, MYH4, MYL1, RYR1, TNNC2, TNNI2, TNNT3, TPM2
		168	1.8	0.027	DOWN: TNNC2, TNNI2, TNNT3 UP: MYL1
	^131^I	24	0.85	–	–
			8.5	<0.0005	DOWN: MEF2C UP: ACTA1, ATP2A1, MYH1, MYH4, MYL1, RYR1, TNNC2, TNNI2, TNNT3, TPM2
			17	*0.071*	*UP: ACTA1, ATP2A1*
Integrin-linked kinase signalling	^211^At	1	0.023	*0.291*	*DOWN: MYL1 UP: DSP, IRS2*
			1.4	<0.0005	DOWN: ACTA1, ACTB, Actn3, CCND1, ITGB6, KRT18, MYH1, MYH2, MYH4, MYL1, VIM UP: IRS2, LIMS2, SH2B2, VIM, ITGB6 UP*/*DOWN: PPP2R5A
		6	0.32	0.004	DOWN: ACTA1, MYH1, MYL1 UP: DSP, IRS2, RHOU
			1.4	0.001	DOWN: PPAP2B UP: ACTA1, ACTN2, Actn3, DSP, MYH1, MYH2, MYH4, RHOU
		24	0.05	*0.203*	*UP: ACTN2, LIMS2, MYH2, RHOT2 UP/DOWN: PPP2R5A*
			0.5	*0.413*	*UP: ACTN2, MYH2*
			1.4	0.001	DOWN: ACTB, CCND1, CDH1, CTNNB1, ITGB4, KRT18 UP: ACTA1, FOS, IRS2, MYH4
			11	<0.0005	DOWN: Irs3, VIM UP: ACTA1, Actn3, CDH1, CREB3L4, DSP, IRS2, ITGB4, ITGB6, MYH1, MYH2, MYH4, MYL1, KRT18
			32	<0.0005	UP: ACTA1, ACTN2, Actn3, CDH1, CREB3L4, DSP, IRS2, ITGB4, ITGB6, KRT18, MYH1, MYH2, MYH4, MYL1
		168	1.8	–	–
	^131^I	24	0.85	*0.169*	*DOWN: ACTB, IRS2, PPAP2B UP: FOS*
			8.5	0.001	DOWN: IRS2 UP: ACTA1, ACTN2, Actn3, CCND1, FOS, MYH1, MYH4, MYL1
			17	*0.076*	*UP: ACTA1, CCND1*
Thyroid cancer signalling	^211^At	1	0.023	*0.291*	*DOWN: MYL1 UP: DSP, IRS2*
			1.4	0.029	DOWN: CCND1, KLK3, NGF UP: PPARG
		6	0.32	0.040	DOWN: NGF DOWN*/*UP: KLK3
			1.4	0.019	UP: NGF, KLK3, RET
		24	0.05	*0.490*	*DOWN/UP: KLK3*
			0.5	0.036	DOWN: KLK3, UP: NGF
			1.4	0.004	DOWN: CCND1, CDH1, CTNNB1 UP: KLK3
			11	<0.0005	DOWN: NTRK2, NTRK3, PPARG UP: CDH1, KLK3, NGF, RET
			32	<0.0005	DOWN: NTRK2, NTRK3 UP: CDH1, KLK3, NGF, RET
		168	1.8	0.026	UP: KLK3, NGF
	^131^I	24	0.85	*0.370*	*UP: PPARG*
			8.5	*0.100*	*UP: CCND1, PPARG,*
			17	0.004	UP: CCND1, KLK3

DOWN and UP indicate down- and upregulation, respectively. Italics indicates no statistically significant effect on the specific canonical pathway

*Abbreviations*: *Δt* exposure time, *D* absorbed dose

^a^IPA predicts involved molecules in the form of human proteins

### Diseases and function analysis: thyroid cancer and disturbed thyroid function

A diseases and function analysis related to thyroid was generated with IPA (Table 3). At 6 (1.4 Gy) and 24 h (11 and 32 Gy) after ^211^At administration, a relation between the transcriptional response and various thyroid cancer types was identified. Such a relation was also identified 24 h after ^131^I administration; however, at a lower absorbed dose level (0.85 and 8.5 Gy). Additionally, the transcriptional response was linked to altered T3 and T4 levels and to thyroid gland development at 0.32 and 32 Gy, 6 and 24 h after ^211^At administration, respectively.

**Table 3:** Results from IPA analysis of diseases and functions related to thyroid after ^131^I and ^211^At exposure

Nuclide	Δ*t* (h)	D (Gy)	Disease or function	*p* value	Involved molecules^a^
^211^At	1	0.023			
		1.4			
	6	0.32	Dystransthyretinemic euthyroidal hyperthyroxinemia	7.92E−03	UP: TTR
			Quantity of L-triiodothyronine	2.65E−03	DOWN: LEP UP: TTR, UCP1
		1.4	Differentiated thyroid cancer	4.63E−04	DOWN: IDH1, MMP2, PDGFRA, RAP1GAP, TEK, TGFBR2 UP: CDKN1A, RET,
			Medullary thyroid cancer	1.01E−03	DOWN: AMY1A (includes others), PDGFRA, TEK UP: RET,
			Thyroid cancer	1.91E−04	DOWN: AMY1A (includes others), ECM1, IDH1, MMP2, PDGFRA, RAP1GAP, SERPINF1, TEK, TGFBR2 UP: CDKN1A, RET
	24	0.05			
		0.5			
		1.4			
		11	Metastasis of thyroid gland tumour	1.21E−03	DOWN: VIM UP: RET
			Thyroid cancer	1.18E−03	DOWN: AMY1A (includes others), NTRK2, PPARG, SLPI, VIM UP: CDH1, PPARGC1A, PRLR, RAP1GAP, RET, SLC5A8, TP63,
		32	Medullary thyroid cancer	1.50E−03	DOWN: AMY1A (includes others), NTRK2 UP: PRLR, RET
			Thyroid gland development	4.71E−03	DOWN: HOXA5, TBX1 UP: RET
	168	1.8			
^131^I	24	0.85	Thyroid cancer	1.58E−04	DOWN: AMY1A (includes others), CDKN1A, ECM1, FLT1, MMP2, PPARGC1A, SERPINF1 UP: PPARG, SPP1, TUBA8
		8.5	Thyroid cancer	2.05E−03	DOWN: AMY1A (includes others), CDKN1A, MMP2, PPARGC1A, PRLR, SERPINF1 UP: CCND1, PPARG, TUBA8
		17	Lack of thyroid gland	9.65E−03	DOWN: FGF10

DOWN and UP indicate down- and upregulation, respectively

*Abbreviations*: *Δt* exposure time, *D* absorbed dose

^a^IPA predicts involved molecules in the form of human proteins

### Upstream regulation of molecules related to peroxisomal proliferator-activated receptors

The upstream regulation analysis generated by IPA predicted upstream regulation of various molecules related to peroxisomal proliferator-activated receptors (PPARs) (Table 4). These included PPARA, PPARD, PPARG, PPARGC1A and several PPAR-targeting drugs and/or PPAR ligands such as GW501516, mono-(2-ethylhexyl)phthalate, pirinixic acid, Rosiglitazone and Troglitazone. IPA predicted that these upstream regulators were generally activated at 1 h, for absorbed dose ≤1.4 Gy at 24 h, and for 1.8 Gy at 168 h.

**Table 4:** Peroxisomal proliferator-activated receptor (PPAR)-related upstream regulators in thyroids exposed to ^131^I or ^211^At according to IPA upstream regulator analysis

Radionuclide	^211^At										^131^I		
Time (h)	1		6		24					168	24		
Injected activity (kBq)	1.7	100	1.7	7.5	0.064	0.64	1.7	14	42	1.7	13	130	260
Absorbed dose (Gy)	0.023	1.4	0.32	1.4	0.05	0.5	1.4	11	32	1.8	0.85	8.5	17
Gene	z score												
Pirinixic acid	3.7	4.7	−0.4	−0.4	5.1	3.6	0.8	−1.4	n.s.	3.5	0.9	1.5	0.2
PPARA	2.7	3.9	−0.6	−1.5	5.1	3.7	2.0	−0.9	1.4	2.8	−0.9	−0.1	−0.7
Troglitazone	2.7	5.0	1.6	−0.3	2.9	2.8	1.1	−2.6	−2.2	1.6	−0.1	−1.2	−2.0
Mono-(2-ethylhexyl)phthalate	2.9	5.6	0.6	0.5	5.0	3.1	1.6	n.s.	n.s.	2.4	0.9	1.6	0.0
PPARG	2.0	5.2	−0.2	−0.9	4.9	2.3	1.8	−1.1	−0.1	2.6	−0.6	1.3	0.0
PPARGC1A	2.2	4.1	−0.4	1.2	4.5	3.0	0.9	0.8	0.8	2.3	0.0	1.8	0.0
Rosiglitazone	1.0	5.1	−0.7	−2.1	4.6	1.2	1.8	−0.9	−0.2	2.0	1.2	0.7	0.0
PPARD	3.1	3.9	1.5	n.s.	3.5	3.7	n.s.	−1.3	n.s.	2.6	−0.8	0.3	n.s.
GW501516	2.6	2.8	0.8	0.8	3.1	3.0	0.0	0.0	0.0	1.8	−1.2	−0.3	0.0

A z score equal to or larger or less than 2 or −2 indicate activated or inhibited upstream regulator, respectively. A z score value between -2 and 2 is considered not statistically significant. n.s. indicates that IPA was not able to predict upstream regulation of that specific upstream regulator for the specific exposure condition

## Discussion

In the present study, the values used to calculate absorbed dose were based on previously published ^131^I and ^211^At biodistribution data, where mice were simultaneously injected with both ^131^I and ^211^At, which allows for direct comparison of the absorbed dose per injected activity between ^131^I and ^211^At in the same animal [26]. In the present study, radioactivity measurements of individual thyroid samples would have enhanced the certainty in absorbed dose calculations but was not possible since all excised thyroid tissue was needed to ensure sufficient amount of RNA for microarray analysis. There are several important differences in the characteristics of ^211^At and ^131^I exposure: i) the difference in mean range of the α and β particles emitted (65 and 400 μm), ii) the much higher mean energy released per decay from ^211^At compared with ^131^I (7000 and 190 keV), iii) the difference in LET of particles emitted from ^131^I and ^211^At (0.25 and 98.8 keV/μm, respectively) and iv) much shorter half-life for ^211^At than ^131^I (7.2 h and 8.0 day, respectively). Taken together, ^211^At irradiates more heterogeneously and with higher dose rates at similar absorbed dose levels compared with ^131^I (and the dose rate will decline faster for ^211^At compared to ^131^I). The effects of radiation quality on global gene expression should be further studied, and to our knowledge, the present study is the first to investigate such differences between ^131^I and ^211^At.

RNA microarray analysis was used to evaluate the impact of ^131^I and ^211^At exposure on global transcriptional regulation in normal mouse thyroid tissue. Regulated genes were associated with biological functions using previously published literature reports and various databases, in addition to upstream and downstream regulation analysis and canonical pathway analysis generated by Ingenuity Pathway Analysis (IPA) software. In total, 1144 genes were differentially regulated showing a large variation in number of genes per group. In general, hierarchical clustering divided groups that received high absorbed doses into one branch and groups receiving low absorbed doses into another. Thus, we hypothesize that the transcriptional profiles presented here may reflect intrinsic biological properties predictive of ^131^I and ^211^At absorbed dose levels at various time points.

At both 1 and 6 h, the number of regulated genes increased with absorbed dose. This was not the case for ^131^I or ^211^At exposure at 24 h, where a broader range of absorbed dose was tested. Furthermore, hierarchical clustering revealed distinct differences between the transcriptional profiles of both similar and different exposures, e.g. the transcriptional profiles for 1.4 Gy at 1 and 6 h after ^211^At administration were distinctly different although the absorbed dose was similar. This could in part be explained by the profound differences in dose rate; dose rate effects on the transcriptional response have previously been described in vivo following radionuclide administration [22, 25, 29]. These findings indicate that variations in the radiation-induced response with absorbed dose will be reflected in the number of regulated genes, in addition to which specific genes are regulated, although not in a clear dose-dependent manner. Instead it is likely that changes in transcriptional patterns in a specific tissue will depend with varying degree on, but not excluded to, the following parameters: exposure time, injected activity, absorbed dose, dose rate, dose distribution (e.g. frequency of non-, single- or multi-hit cells) and radiation quality. Each unique setup of these parameters may then yield a specific response in the target tissue. In addition, cells are dynamic systems with complex regulatory networks that activate cascades of downstream regulation that is sensitive to type and frequency of incoming stimulus.

It is valuable to identify genes with exposure-specific expression as they may be used as biomarkers. Biomarkers are useful to better understand the mechanisms behind the radiation-induced response. A potential application of biomarkers for ionizing radiation exposure of the thyroid might be in biological dosimetry after exposure to relatively high doses, maybe in a triage setting. In the present study, kallikrein 1 (*Klk1*) and 12 of 13 kallikrein 1-related (*Klk1b*) peptidases in the mouse genome were frequently regulated with fold change values between −3.8 and 110. The expression of these genes generally increased with absorbed dose and time after injection of ^131^I or ^211^At; however, 32 Gy resulted in less upregulation compared with 11 Gy (24 h, ^211^At) and the highest dose rate used (1.4 Gy, 1 h, ^211^At) resulted in downregulation. It is likely that the expression of *Klk1* and *Klk1*-related peptidases depends, to a different degree, on dose rate, absorbed dose and time after injection. In a study on the rat urine proteome 24 h after 10 Gy total body irradiation, the occurrence of kallikrein 1-related peptidase b24 precursor protein increased while the kallikrein-binding serine protease inhibitor A3K precursor decreased [30]. In another study, the plasma kallikrein levels decreased with absorbed dose (0–19 Gy) at 2–24 h after local irradiation of the hind legs in tumour-bearing rats and controls [31]. Additionally, we have shown that regulation of *Klk1* and *Klk1*-related genes in mouse thyroids after ^131^I exposure does not show a circadian variation [32]. We hypothesize that genes involved in the kallikrein network may be potential biomarkers of radiation exposure, but further research is warranted to elucidate the relationship between radiation exposure and kallikrein proteases and kallikrein inhibitor levels. The kallikrein genes have also been shown to contribute to the radiation-induced death of various species. After treatment with soy bean trypsin inhibitors (SBTI), the mortality rate in mice and chickens 14 days after exposure to 690 and 820 R (6.7 and 8 Gy to soft tissue), respectively, decreased from 100 to 50 % in mice and from 86 to 4 % in chickens [33]. The authors suggested that the decrease in mortality rate after administration of SBTI originated from a radioprotective effect on the vascular system with less vascular leakage and that the protease inhibited was likely tissue pre-kallikrein. We suggest that the radioprotective role of SBTI should be further assessed.

Recurrently regulated genes might be potential biomarkers and show how different exposure types influence similar/related genes and biological functions, although maybe with different magnitude and/or direction of regulation. The 27 recurrently regulated genes in the present study were divided into six clusters according to the transcriptional pattern of each individual gene following a specific exposure. In cluster 1, ^211^At-induced regulation was dependent on both absorbed dose and time after exposure with monotonous change in regulation at 24 h, and 8.5 Gy ^131^I exposure resulted in very high upregulation. Genes in cluster 1 are related to muscular activity and/or calcium activity (*Atp2a1*, *Eno3*, *Pvalb*, *Tnnc2*, *Tnni2* and *Tnnt3*). Notably, the thyroid gland contains parafollicular cells (C-cells) that produce the calcium homeostasis regulating hormone calcitonin. Cluster 2 contains genes related to various biological functions, e.g. cellular and tissue development and wound healing (*Ctgf*) [34], energy transduction (*Coq10b*) and circadian rhythm (*Dbp*, *Per1*) [35, 36]. These genes were generally upregulated and may be indicators of radiation exposure in general. Cluster 3 consisted of only one gene (*Mfsd2*) that was up- and downregulated after ^211^At and ^131^I exposure, respectively, indicating a difference between radiation qualities (*Ctgf*, *Per1*, *S100a8*, *S100a9*, also showed a radiation quality dependency). No clear connection to the immune system, inflammation or the cytokine system was found for genes in clusters 1–3. However, the sole gene in cluster 4 (*Ltf*), all genes in cluster 5 (*Ccl8*, *Ly6g6d*, *S100a8*, *S100a9*) and a majority of genes in cluster 6 (*Aoc3*, *Ccl9*, *Clec2d*, *Cpa3*, *Fstl1*, *Scara3*) were related to the immune system in various ways, and many related to both inflammation and the cytokine network [37–47]. The cytokine encoded by *Ccl9* is associated with systemic inflammation and has increased expression in macrophages after exposure to triiodothyronine [43]. Lactoferrin—in mice encoded by the *Ltf* gene, solely expressed in cluster 4 and generally downregulated—has been patented as a radioprotective drug and increased survival in mice exposed to 10 Gy (whole-body, external irradiation) via an impact on, e.g., cytokine regulation [38]. In cluster 5, genes were oppositely regulated when comparing ^131^I and ^211^At exposure, indicating a radiation quality-dependent immune response. In cluster 6, genes were downregulated at low absorbed doses and upregulated at high absorbed dose levels even though the shift from down- to upregulation occurred at a lower absorbed dose level for ^211^At compared with ^131^I. This suggests that the radiation-induced regulation of genes in cluster 6 is dependent on both radiation quality and absorbed dose. The 27 recurrently regulated genes can potentially be used to discriminate between several different exposure parameters, e.g. radiation quality, absorbed dose levels and time after administration, and might be considered as potential biomarkers for at least ^131^I and ^211^At exposure of thyroid. These results indicate a connection between specific exposures and biological responses, especially for the genes in clusters 4–6 that were clearly associated with immunological response, inflammation and the cytokine network. These recurrently regulated genes should be further studied to better understand their impact on radiation-induced biological responses and in particular the local and systemic effects that involve inflammation, the immune system and the cytokine network.

To assess systemic effects from ^131^I and ^211^At exposure, regulation of the 27 recurring genes was compared with transcriptional changes in the lungs, spleen, liver and kidney cortex and medulla in the same mice dissected in the present study [23, 24]. These non-thyroidal tissues, that are exposed at a much lower absorbed dose level compared with thyroid, shared regulation of 19/27 and 6/27 recurring genes after ^211^At and ^131^I exposure, respectively. Additionally, we have previously shown that the transcriptional response in the lungs, spleen, liver and kidney cortex and medulla in mice administered ^131^I and ^211^At can partly be explained as a systemic response from radiation-induced effects on thyroid [32]. One gene with potential biomarker properties is *Dbp*. The *Dbp* gene expression pattern changed in several non-thyroidal tissues after low absorbed dose level exposure to both ^131^I and ^211^At [23, 24], in kidneys in mice both early and late after ^177^Lu-octreotate administration [48], and in rat thyroids after ^131^I administration [49].

A connection to thyroid cancer was detected for 5/27 recurring genes. PVALB has been suggested as an ideal biomarker to discriminate between benign and malignant thyroid cancer [50]. *ENO3* is another cancer-related gene, associated to the PAX8-PPARG fusion protein in thyroid follicular carcinomas, and upstream regulation of PPARs and PPAR-related pathways was detected in the present study [51]. The level of CTGF correlated with metastasis, tumour size and clinical stage for papillary thyroid carcinoma in a previous study [52]. Furthermore, undifferentiated thyroid carcinomas have been shown to be S100A8/9 immunopositive and both genes were associated with, e.g., inflammation-associated cancer and aggressive breast cancer [41, 53, 54].

According to the Ingenuity canonical pathway and diseases and functions analysis tool, the transcriptional response after both ^131^I and ^211^At exposure was related to thyroid cancer signalling and various thyroid cancers, respectively. It is uncertain whether induction of thyroid cancer can be detected at the transcriptional level at these early times after initiation of radiation exposure. However, according to IPA, exposure to ionizing radiation activates thyroid cancer signalling by rearrangements of *RET* and/or *NTRK*, both present in some of the exposed groups. Unfortunately, all parts of the thyroid samples from mice in the three studies this work is based on were used for microarray analysis, why further studies of genomic rearrangements were not possible from the same samples.

Additionally, KLK3 (human denotation of KLK1 in mouse) is among the involved molecules in all groups that show an impact on thyroid cancer signalling. Since IPA uses human protein nomenclature for annotation of genes, the presence of KLK3 in the IPA analysis is likely a result of regulation of mouse *Klk1* and *Klk1*-related genes in the present study.

The number of annotated genes involved in calcium signalling generally increased with absorbed dose for ^211^At exposure. Several of the genes associated with these molecules could be found in cluster 1 among the 27 recurrently regulated genes (*Tnni2*, *Tnnc2*, *Tnnt3* and *Atp2a1*). Additionally, gene products of several other recurrently regulated genes are also calcium-related according to literature reports, but not associated with calcium signalling in the IPA canonical pathway analysis. For example, the gene products of *Clec2d* and *Ogn* both inhibit osteoclasts that can release Ca^2+^ into the blood [55, 56]. As previously mentioned, the thyroid gland also contains parafollicular cells that produce calcitonin, a hormone partly responsible for calcium homeostasis and an inhibitor of osteoclast activity. However, the impact on calcitonin levels from ^131^I and ^211^At exposure was not investigated in the present study. A relationship between calcium and radiation-induced response has been previously reported and it was shown that calcium was required for bystander-induced apoptosis in unirradiated keratinocytes [57].

An impact on integrin-linked kinase (ILK) signalling was identified using an IPA canonical pathway analysis, and some genes associated with calcium signalling were also associated with ILK signalling in the present study. For ^211^At, a higher absorbed dose generally resulted in a response involving a higher number of annotated genes. For ^131^I exposure, ILK signalling was only statistically significant after 8.5 Gy, suggesting a difference in response due to radiation quality. In blood from mice administered with ^137^Cs, genes associated with integrin-signalling were found upregulated at days 2 and 3 and downregulated at days 20 and 30 (transcriptional level) [58]. No clear temporal effect on integrin-signalling was seen during the somewhat shorter time range used in the present study. Interestingly, however, in the present study, we demonstrate that genes, e.g., associated with ILK signalling were generally upregulated at higher absorbed dose levels and downregulated at lower absorbed dose levels, suggesting different involvement of this signalling pathway at different absorbed dose levels. This was especially the case 24 h after ^211^At administration, but a similar trend was also seen after ^131^I administration. ILK signalling and radiation damage have been previously connected, and furthermore, ILK signalling partly controls cell adhesion and mediates prosurvival and antiapoptotic signalling after exposure to ionizing radiation [59]. Additionally, several cell adhesion GO terms were identified when performing in-depth separate analysis of the transcriptional response of thyroid tissue from animals in the three experiments that constitute the present study [20–22].

In the present study, the predicted upstream regulation of several peroxisomal proliferator-activated receptors (PPARs), and PPAR ligands and agonists was found. The PPARs are of interest in the radiation-induced biological response. In one study, administration of the PPARα agonist fenofibrate prevented some cognitive function impairment in young rats exposed to 40 Gy fractionated whole-brain irradiation [60]. In another study, knockout of PPARα resulted in inhibition of radiation-induced apoptosis in the mouse kidney through regulation of *Nfkb* and anti-apoptosis factors [61]. Together, these results indicate that the PPAR network may play a role in radiation-induced biological response and that it may be targeted to modulate radiation damage in various tissues.

## Conclusions

A profound effect on gene expression in mouse thyroid tissue after ^131^I and ^211^At exposure was detected, and 27 genes, of which many are associated with immune response, were identified as potential biomarkers for ^131^I and ^211^At exposure, and the biomarker applicability of these genes should be further studied. Hierarchical clustering revealed distinct differences between transcriptional profiles of both similar and different exposures, demonstrating the necessity for better understanding of radiation-induced changes in cellular activity. Additionally, the kallikrein network deserves further attention since literature shows how administration of protease inhibitors, known to decrease kallikrein levels, drastically increased survival in various irradiated species. An effect on thyroid cancer signalling was identified, as well as regulation of several genes previously identified as biomarkers for thyroid cancer; however, it is unlikely that thyroid cancer can be manifested at the transcriptional level at these early times after initiation of radiation exposure. Furthermore, the present study supports that ionizing radiation may impact calcium-related biological processes. Taken together, we consider RNA microarrays, especially in the in vivo setting, to be an important tool to gain further insight in the complex radiation-induced changes in cellular activity and affected pathways, and for biomarker discovery.

### Compliance with ethical standards

All applicable international, national, and/or institutional guidelines for the care and use of animals were followed. The study design was approved by the Ethical Committee on Animal Experiments in Gothenburg, Sweden. This article does not contain any studies with human participants performed by any of the authors.

## References

[1] Cooper DS, Doherty GM, Haugen BR, Kloos RT, Lee SL, Mandel SJ (2009). Revised American Thyroid Association management guidelines for patients with thyroid nodules and differentiated thyroid cancer: the American Thyroid Association (ATA) guidelines taskforce on thyroid nodules and differentiated thyroid cancer. Thyroid.

[2] Kaminski MS, Tuck M, Estes J, Kolstad A, Ross CW, Zasadny K (2005). 131I-tositumomab therapy as initial treatment for follicular lymphoma. New Engl J Med.

[3] Bombardieri E, Giammarile F, Aktolun C, Baum RP, Delaloye AB, Maffioli L (2010). 131I/123I-metaiodobenzylguanidine (mIBG) scintigraphy: procedure guidelines for tumour imaging. EJNMMI.

[4] Reid JR, Wheeler SF (2005). Hyperthyroidism: diagnosis and treatment. Am Fam Physician.

[5] Brown I. Astatine-211: its possible applications in cancer therapy. Int J Rad Appl Instrum A. 1986;37(8):789–98. 10.1016/0883-2889(86)90273-x 3021680

[6] Andersson H, Cederkrantz E, Back T, Divgi C, Elgqvist J, Himmelman J (2009). Intraperitoneal alpha-particle radioimmunotherapy of ovarian cancer patients: pharmacokinetics and dosimetry of (211)At-MX35 F(ab’)2--a phase I study. J Nucl Med.

[7] Vaidyanathan G, Zalutsky MR (2011). Applications of 211At and 223Ra in targeted alpha-particle radiotherapy. Curr Radiopharm.

[8] Zalutsky MR, Reardon DA, Pozzi OR, Vaidyanathan G, Bigner DD (2007). Targeted alpha-particle radiotherapy with 211At-labeled monoclonal antibodies. Nucl Med Biol.

[9] Spetz J, Rudqvist N, Forssell-Aronsson E (2013). Biodistribution and dosimetry of free 211At, 125I− and 131I− in rats. Cancer Biother Radiopharm.

[10] Lundh C, Lindencrona U, Schmitt A, Nilsson M, Forssell-Aronsson E (2006). Biodistribution of free 211At and 125I-in nude mice bearing tumors derived from anaplastic thyroid carcinoma cell lines. Cancer Biother Radiopharm.

[11] Lindencrona U, Nilsson M, Forssell-Aronsson E. Similarities and differences between free 211 At and 125I− transport in porcine thyroid epithelial cells cultured in bicameral chambers. Nucl Med Biol. 2001;28(1):41–50. 10.1016/s0969-8051(00)00179-7 11182563

[12] Brans B, Monsieurs M, Laureys G, Kaufman JM, Thierens H, Dierckx RA (2002). Thyroidal uptake and radiation dose after repetitive I-131-MIBG treatments: influence of potassium iodide for thyroid blocking. Med Pediatr Oncol.

[13] Annex J (2000). Exposures and effects of the Chernobyl accident. Sources and Effects of Ionizing Radiation: The United Nations Scientific Committee on the Effects of Atomic Radiation UNSCEAR.

[14] Cardis E, Kesminiene A, Ivanov V, Malakhova I, Shibata Y, Khrouch V (2005). Risk of thyroid cancer after exposure to 131I in childhood. JNCI.

[15] Danielsson A, Claesson K, Parris TZ, Helou K, Nemes S, Elmroth K (2013). Differential gene expression in human fibroblasts after alpha-particle emitter (211)At compared with (60)Co irradiation. Int J Radiat Biol.

[16] Seidl C, Port M, Apostolidis C, Bruchertseifer F, Schwaiger M, Senekowitsch-Schmidtke R (2010). Differential gene expression triggered by highly cytotoxic alpha-emitter-immunoconjugates in gastric cancer cells. Investig New Drugs.

[17] Seidl C, Port M, Gilbertz KP, Morgenstern A, Bruchertseifer F, Schwaiger M (2007). 213Bi-induced death of HSC45-M2 gastric cancer cells is characterized by G2 arrest and up-regulation of genes known to prevent apoptosis but induce necrosis and mitotic catastrophe. Mol Cancer Ther.

[18] Yong KJ, Milenic DE, Baidoo KE, Brechbiel MW (2014). Impact of alpha-targeted radiation therapy on gene expression in a pre-clinical model for disseminated peritoneal disease when combined with paclitaxel. PLoS ONE.

[19] Yong KJ, Milenic DE, Baidoo KE, Kim YS, Brechbiel MW (2013). Gene expression profiling upon (212) Pb-TCMC-trastuzumab treatment in the LS-174T i.p. xenograft model. Cancer Med.

[20] Rudqvist N, Parris TZ, Schuler E, Helou K, Forssell-Aronsson E (2012). Transcriptional response of BALB/c mouse thyroids following in vivo astatine-211 exposure reveals distinct gene expression profiles. EJNMMI Res.

[21] Rudqvist N, Schüler E, Parris TZ, Langen B, Helou K, Forssell-Aronsson E. Dose-specific transcriptional responses in thyroid tissue in mice after 131I administration. Nuclear Med and Biol. 2014; 42(3): 263-268. doi:10.1016/j.nucmedbio.2014.11.006. 10.1016/j.nucmedbio.2014.11.006 25496975

[22] Rudqvist N, Spetz J, Schüler E, Parris TZ, Langen B, Helou K (2015). Transcriptional response in mouse thyroid tissue after 211 at administration: effects of absorbed dose, initial dose-rate and time after administration. PLoS ONE.

[23] Schuler E, Parris TZ, Rudqvist N, Helou K, Forssell-Aronsson E (2011). Effects of internal low-dose irradiation from 131I on gene expression in normal tissues in Balb/c mice. EJNMMI Res.

[24] Langen B, Rudqvist N, Parris TZ, Schuler E, Helou K, Forssell-Aronsson E (2013). Comparative analysis of transcriptional gene regulation indicates similar physiologic response in mouse tissues at low absorbed doses from intravenously administered 211At. J Nucl Med.

[25] Langen B, Rudqvist N, Parris TZ, Schüler E, Spetz J, Helou K (2015). Transcriptional response in normal mouse tissues after iv 211At administration-response related to absorbed dose, dose rate, and time. EJNMMI Res.

[26] Garg PK, Harrison CL, Zalutsky MR (1990). Comparative tissue distribution in mice of the alpha-emitter 211At and 131I as labels of a monoclonal antibody and F(ab’)2 fragment. Cancer Res.

[27] Flynn AA, Green AJ, Pedley RB, Boxer GM, Boden R, Begent RH (2001). A mouse model for calculating the absorbed beta-particle dose from (131)I- and (90)Y-labeled immunoconjugates, including a method for dealing with heterogeneity in kidney and tumor. Radiat Res.

[28] Lance GN, Williams WT (1967). A general theory of classificatory sorting strategies II. Clustering systems. Comput J.

[29] Schuler E, Rudqvist N, Parris TZ, Langen B, Spetz J, Helou K (2014). Time- and dose rate-related effects of internal (177)Lu exposure on gene expression in mouse kidney tissue. Nucl Med Biol.

[30] Sharma M, Halligan BD, Wakim BT, Savin VJ, Cohen EP, Moulder JE (2008). The urine proteome as a biomarker of radiation injury: submitted to proteomics- clinical applications special issue: “renal and urinary proteomics (Thongboonkerd)”. Proteomics Clin Appl.

[31] Makoyo PO, West WL (1977). Effect of focal irradiation on plasma kallikrein activity in tumor bearing rats. Int J Radiat Oncol Biol Phys.

[32] Langen B. Systemic effects after ionizing radiation exposure: Genome-wide transcriptional analysis of mouse normal tissues exposed to (211) At,(131) I, or 4 MV photon beam: PhD thesis, Chalmers University of Technology, Gothenburg, Sweden. 2015.

[33] Palladino MA, Galton JE, Troll W, Thorbecke GJ (1982). Gamma-irradiation-induced mortality: protective effect of protease inhibitors in chickens and mice. Int J Radiat Biol Relat Stud Phys Chem Med.

[34] Hall-Glenn F, Lyons KM (2011). Roles for CCN2 in normal physiological processes. Cell Mol Life Sci.

[35] Curie T, Mongrain V, Dorsaz S, Mang GM, Emmenegger Y, Franken P (2013). Homeostatic and circadian contribution to EEG and molecular state variables of sleep regulation. Sleep.

[36] Sun ZS, Albrecht U, Zhuchenko O, Bailey J, Eichele G, Lee CC (1997). RIGUI, a putative mammalian ortholog of the Drosophila period gene. Cell.

[37] Sanchez L, Calvo M, Brock JH (1992). Biological role of lactoferrin. Arch Dis Child.

[38] Varadhachary A, Petrak K, Blezinger P, inventors; Google Patents, assignee (2010). Lactoferrin as a radioprotective agent.

[39] Muller K, Meineke V (2011). Radiation-induced mast cell mediators differentially modulate chemokine release from dermal fibroblasts. J Dermatol Sci.

[40] Mallya M, Campbell RD, Aguado B (2002). Transcriptional analysis of a novel cluster of LY-6 family members in the human and mouse major histocompatibility complex: five genes with many splice forms. Genomics.

[41] Gebhardt C, Nemeth J, Angel P, Hess J (2006). S100A8 and S100A9 in inflammation and cancer. Biochem Pharmacol.

[42] Smith DJ, Salmi M, Bono P, Hellman J, Leu T, Jalkanen S (1998). Cloning of vascular adhesion protein 1 reveals a novel multifunctional adhesion molecule. J Exp Med.

[43] Perrotta C, Buldorini M, Assi E, Cazzato D, De Palma C, Clementi E (2014). The thyroid hormone triiodothyronine controls macrophage maturation and functions: protective role during inflammation. Am J Pathol.

[44] Carlyle JR, Jamieson AM, Gasser S, Clingan CS, Arase H, Raulet DH (2004). Missing self-recognition of Ocil/Clr-b by inhibitory NKR-P1 natural killer cell receptors. Proc Natl Acad Sci U S A.

[45] Pejler G, Knight SD, Henningsson F, Wernersson S (2009). Novel insights into the biological function of mast cell carboxypeptidase A. Trends Immunol.

[46] Chaly Y, Fu Y, Marinov A, Hostager B, Yan W, Campfield B (2014). Follistatin-like protein 1 enhances NLRP3 inflammasome-mediated IL-1beta secretion from monocytes and macrophages. Eur J Immunol.

[47] Han HJ, Tokino T, Nakamura Y (1998). CSR, a scavenger receptor-like protein with a protective role against cellular damage causedby UV irradiation and oxidative stress. Hum Mol Genet.

[48] Schuler E. Biomarker discovery and assessment for prediction of kidney response after 177Lu-octreotate therapy. PhD thesis, Department of Radiation Physics, Institution of Clinical Sciences, Sahlgrenska Academy, University of Gothenburg. 2014.

[49] Rudqvist N. Radiobiological effects of the thyroid gland: Transcriptomic and proteomic responses to 131I and 211At exposure: PhD thesis, Department of Radiation Physics, Institution of Clinical Sciences, Sahlgrenska Academy, University of Gothenburg, Gothenburg, Sweden, 2015.

[50] Cerutti JM, Oler G, Delcelo R, Gerardt R, Michaluart P, de Souza SJ (2011). PVALB, a new Hurthle adenoma diagnostic marker identified through gene expression. J Clin Endocrinol Metab.

[51] Giordano TJ, Au AY, Kuick R, Thomas DG, Rhodes DR, Wilhelm KG (2006). Delineation, functional validation, and bioinformatic evaluation of gene expression in thyroid follicular carcinomas with the PAX8-PPARG translocation. Clin Cancer Res.

[52] Cui L, Zhang Q, Mao Z, Chen J, Wang X, Qu J (2011). CTGF is overexpressed in papillary thyroid carcinoma and promotes the growth of papillary thyroid cancer cells. Tumour Biol.

[53] Ito Y, Arai K, Nozawa R, Yoshida H, Hirokawa M, Fukushima M (2009). S100A8 and S100A9 expression is a crucial factor for dedifferentiation in thyroid carcinoma. Anticancer Res.

[54] Parris TZ, Kovacs A, Aziz L, Hajizadeh S, Nemes S, Semaan M (2014). Additive effect of the AZGP1, PIP, S100A8 and UBE2C molecular biomarkers improves outcome prediction in breast carcinoma. Int J Cancer.

[55] Kukita A, Bonewald L, Rosen D, Seyedin S, Mundy GR, Roodman GD (1990). Osteoinductive factor inhibits formation of human osteoclast-like cells. Proc Natl Acad Sci U S A.

[56] Hu YS, Zhou H, Myers D, Quinn JM, Atkins GJ, Ly C (2004). Isolation of a human homolog of osteoclast inhibitory lectin that inhibits the formation and function of osteoclasts. J Bone Miner Res.

[57] Lyng FM, Maguire P, McClean B, Seymour C, Mothersill C (2006). The involvement of calcium and MAP kinase signaling pathways in the production of radiation-induced bystander effects. Radiat Res.

[58] Paul S, Ghandhi SA, Weber W, Doyle-Eisele M, Melo D, Guilmette R (2014). Gene expression response of mice after a single dose of 137CS as an internal emitter. Radiat Res.

[59] Cordes N (2004). Overexpression of hyperactive integrin-linked kinase leads to increased cellular radiosensitivity. Cancer Res.

[60] Greene-Schloesser D, Payne V, Peiffer AM, Hsu FC, Riddle DR, Zhao W (2014). The peroxisomal proliferator-activated receptor (PPAR) alpha agonist, fenofibrate, prevents fractionated whole-brain irradiation-induced cognitive impairment. Radiat Res.

[61] Zhao W, Iskandar S, Kooshki M, Sharpe JG, Payne V, Robbins ME (2007). Knocking out peroxisome proliferator-activated receptor (PPAR) alpha inhibits radiation-induced apoptosis in the mouse kidney through activation of NF-kappaB and increased expression of IAPs. Radiat Res.

